# Autologous Adipocyte Derived Stem Cells Favour Healing in a Minipig Model of Cutaneous Radiation Syndrome

**DOI:** 10.1371/journal.pone.0031694

**Published:** 2012-02-14

**Authors:** Fabien Forcheron, Diane Agay, Harry Scherthan, Diane Riccobono, Francis Herodin, Viktor Meineke, Michel Drouet

**Affiliations:** 1 Institut de Recherche Biomédicale des Armées-antenne La Tronche, La Tronche, France; 2 Institut für Radiobiologie der Bundeswehr, München, Germany; University Health Network, Canada

## Abstract

Cutaneous radiation syndrome (CRS) is the delayed consequence of localized skin exposure to high doses of ionizing radiation. Here we examined for the first time in a large animal model the therapeutic potential of autologous adipose tissue-derived stroma cells (ASCs). For experiments, Göttingen minipigs were locally gamma irradiated using a ^60^Co source at the dose of 50 Gy and grafted (n = 5) or not (n = 8). ASCs were cultured in MEM-alpha with 10% fetal calf serum and basic fibroblast growth factor (2 ng.mL^−1^) and post irradiation were intradermally injected on days 25, 46, 67 and finally between days 95 and 115 (50×10^6^ ASCs each time) into the exposed area. All controls exhibited a clinical evolution with final necrosis (day 91). In grafted pigs an ultimate wound healing was observed in four out of five grafted animals (day 130 +/− 28). Immunohistological analysis of cytokeratin expression showed a complete epidermis recovery. Grafted ASCs accumulated at the dermis/subcutis barrier in which they attracted numerous immune cells, and even an increased vasculature in one pig. Globally this study suggests that local injection of ASCs may represent a useful strategy to mitigate CRS.

## Introduction

Skin is a radiosensitive tissue which is exposed to significant radiation doses in radiotherapy and radio-oncology as are the underlying muscles, nerves and vasculature structures [1]. Intentional or accidental exposure of a relatively large area as well as localized “hot spot” exposures of the skin lead to severe damage of many of its cellular components and to the cutaneous radiation syndrome (CRS). The pathophysiology includes radiation-induced cell death (epidermal stem cell depletion, loss of capillary integrity) as well as impairment of the complex communication network between keratinocytes, dermal fibroblasts and resident or circulating immuno-competent cells. The classical clinical evolution of CRS is characterized by the delayed onset of the manifestation stage and consists in transient early erythema, dry/moist desquamation, dermal ischemia/necrosis and dermal atrophy [2]. The severity of the CRS depends on the radiation dose, quality and depth of penetration. Irradiation severely impairs wound healing which normally requires a well-orchestrated integration of complex biological and molecular events, including cell migration and proliferation, extracellular matrix deposition, angiogenesis and remodelling. Nowadays the treatment schedules for severe radiation burns are complex, and include excision of the highly exposed necrotic areas (likely >20 Gy) followed by transient coverage of the wound bed and then by autologous skin grafts.

It has been hypothesized for some years that stem cell injection could reduce normal tissue injury or assists its recovery after irradiation [3], [4] but the role of local trophic secretions, anti-inflammatory activity and putative plasticity [5] have to be studied in more detail. Thus mesenchymal stem cells contained in the stromal vascular fraction of adipose tissues (ASCs) [6], [7] may represent a new promising multipotent stem cell source.

Using a previously described minipig model [8], we here demonstrate for the first time in a large animal model the therapeutic potential of iterative autologous ASC grafting following high dose local irradiation. Wound healing, involving vascular remodelling and immunocompetent cell recruitment, was accelerated in 4 out of 5 treated animals, whereas all PBS-injected controls exhibited a severe necrotic process.

## Materials and Methods

### Animals

Thirteen female Göttingen Minipigs® weighing about 20 kg were purchased from Ellegaard (Dalmose, Denmark) and housed in individual pen (21±1°C, 55% relative humidity, 12 h/12 h light-dark schedule) where they received solid food twice a day and had access to water *ad libitum*.

### Ethical treatment of animals

This study was carried out in strict accordance with the European legislation for the care of laboratory animals. The protocol was approved by the French Army Animal Ethics Committee (Permit number: 2008/24.0). The irradiation of the animals and all cell engraftments were performed under anesthesia, and all efforts were made during the study to minimize suffering of the minipigs.

### ASC harvest and culture

One month prior irradiation, subcutaneous adipose tissue was collected under anaesthesia from the animal chest. Fat tissue was minced and incubated for 60 min at 37°C with collagenase II (Worthington, Lakewood, New Jersey, US). After centrifugation and filtration, adherent fraction from pelleted cells was cultured in minimum essential medium (MEMα) (Invitrogen Life Technologies, Carlsbad, New Mexico, US) supplemented with 10% fetal calf serum (HyClone part of Thermo Fisher Scientific, Waltham, Massachussetts, US) and basic fibroblast growth factor (2 ng.mL^−1^) at 37°C in air with 5% CO_2_. Passages 3 to 8 ASCs were characterized at the end of the culture phase to control stemness using flow cytometry (BD LSRII, BD Biosciences, Pont de Claix, France) and their differentiation potential was assessed [8].

### ASC transplantation in irradiated minipigs

The exposure to a collimated ^60^Co gamma beam (IRDI4000, Alstom, Levallois-Perret, France) of the anesthetized animals induced a delayed burn injury limited to a narrow 20–25 cm long rectangle of lumbar skin and superficial underlying muscles, perpendicular to the body axis, without any vital peritoneal organ irradiation [8]. The dosimetric evaluation indicated a homogeneous dose of 51.4±5.0 Gy in the entry area, ranging to 14.3±2.5 Gy in the exit area.

**Figure 1:**
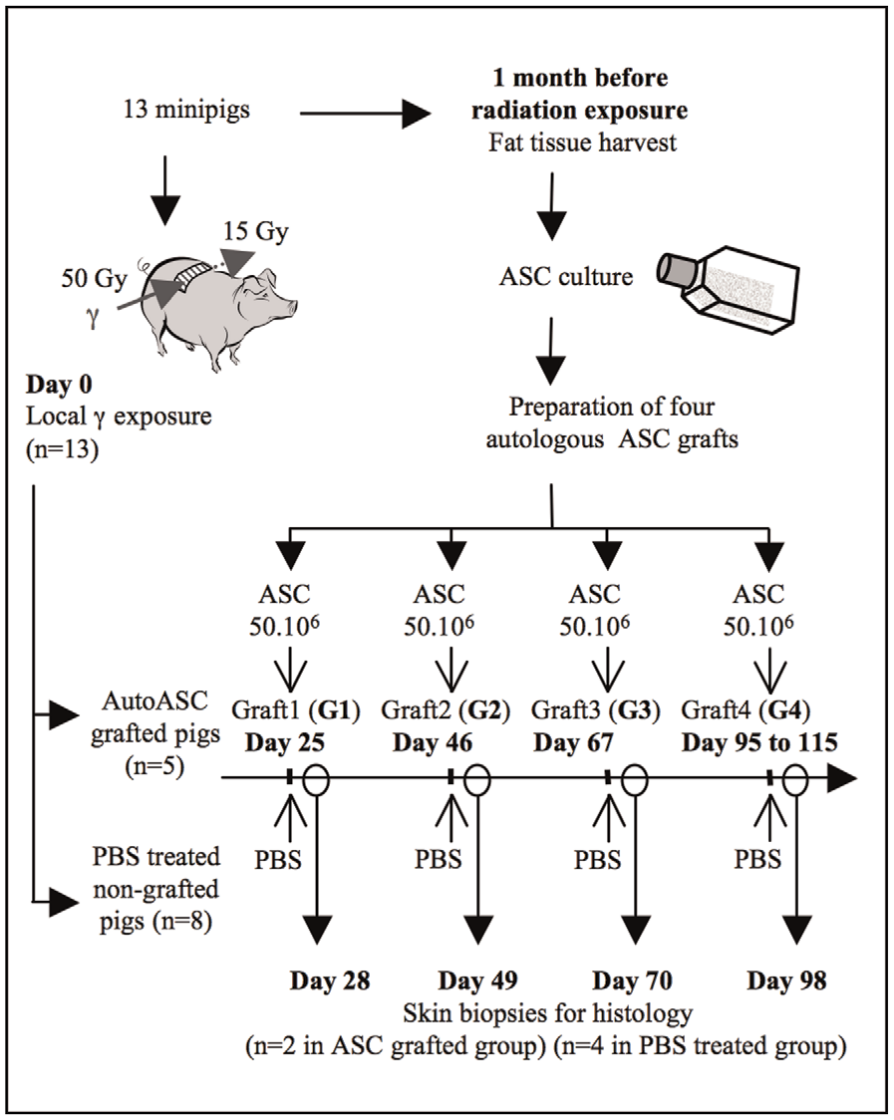
Study design.

The thirteen irradiated animals were randomly divided into two groups (Fig. 1). ASC-grafted minipigs (n = 5) were intradermally injected with autologous ASCs, four times over a 3 month period following irradiation (on days 25 and 46, before the onset of clinical symptoms, then 67, and finally between days 95 and 115). Each grafted minipig received a total of about 200×10^6^ ASCs, corresponding to four grafts of 50×10^6^ ASCs each time distributed in 32×10^6^ ASCs throw the entry area exposed to the major dose of 50 Gy where quick necrosis was predictable (1×10^6^ ASCs in 100 µl PBS in every cm^2^ of damaged skin) and in 18×10^6^ ASCs in the less irradiated exit area where pathological evolution was expected less severe (1×10^6^ ASCs in 100 µl PBS in every 2 cm^2^ of skin). Non-grafted controls (n = 8) were injected with PBS alone according to the same scheme.

### Clinical follow-up and sample harvest

During the follow-up period, all minipigs received a clinical observation three times a week in order to establish a clinical score including all pathological signs and pain evaluation. The initial score of healthy skin was 10, and a negative or a positive point was recorded for each clinical sign. A negative point was recorded for each sign as erythema, subcutaneous oedema, induration, formation of a purple spot corresponding to circulatory trouble, dry skin desquamation, wet peeling, partial or complete crust over the irradiated surface (from −1 to −3 points according to the spread), signs of necrosis (−2 points). To establish whether or not analgesics were needed, a veterinary examination was performed every day with evaluation of the overall condition of the animal based on drink and food intakes and behaviour, on local examination and palpation of the lesions and on assessment of analgesic treatment efficiency. Additive negative points were local sensitivity, spontaneous pain without contact solicitation, pain resistance to major analgesics (Fentanyl 50 µg.h^−1^, Durogesic™ 8.4 mg.21 cm^−2^, Janssen-Cilag, France). A positive point was recorded for the vanishing of previously described signs, for pain relief, for particularly well being, and for the complete healing of radio-induced burn injuries with re-epidermalization of the wound surface. Potential tissue inflammatory processes were assessed at weekly intervals by using a thermal camera (Thermacam, Flir Systems, Issy-les-Moulineaux, France) and by measuring the skin dielectric constant which reflects the cutaneous fluid content (MoistureMeter D probe M25, Delfin Technologies, Kuopio, Finland) [9]. The end point of the experiment was defined as definite extensive skin necrosis not evolving towards healing (failure) or complete healing with epidermal regeneration (success). At this time, the pigs were killed (Dolethal, Vétoquinol SA, Lure, France) and underwent a delayed histological analysis. In addition, a kinetic study was performed in 4 controls and 2 grafted minipigs in which skin biopsies were harvested (biopunch 4 mm diameter, Kruuse, North Yorkshire, UK) from irradiated (entry) and time-matched non-irradiated skin area, three days after each ASC graft or PBS injection. The samples were immediately conditioned for histological analysis.

### Histological procedures

For histological analysis, tissue samples were recovered by punch biopsy and immediately submersed in 4% formaldehyde/PBS (pH 7) overnight at room temperature. Biopsies were washed three times 5 min in PBS/0.1% Glycin and once in 0.9% NaCl at room temperature, subsequently dehydrated through a graded ethanol series and finally incubated for 30 min in toluene. Thereafter, tissue was embedded in Paraffin (Carl Roth, Karlsruhe,Germany) according to standard procedures.

For immunofluorescent staining, paraffin tissue sections were obtained at 5 µm thickness with a 2055 microtome (Leica, Wetzlar, Germany). Immunofluorescent staining was performed on deparaffinized and rehydrated sections subjected to antigen unmasking using 10 mM citrate buffer (pH 6) for 1 h at 95°C. After 3×5 min washes in PBS, sections were extracted for 20 min with 0.5% Triton X-100 in PBS on ice followed by 3×5 min washes in PBTC (PBS, 0.1% Na-Casein, 0.05% Tween-20, 0.1% fish gelatin) and incubated with the primary antibodies (DakoCytomation, Hamburg, Germany) over night at 4°C: monoclonal mouse anti-Ki67 (M7240 diluted to 1/250) for staining of proliferating cell nuclei, rabbit anti CD3 (N1580 used undiluted) and rabbit anti lambda light chain (A0193 diluted to 1/500) for lymphocyte labeling, mouse anti pan-cytokeratin AE1/AE3 (M3515 diluted to 1/200) for pan-cytokeratin staining, and rabbit anti-human von Willebrand factor (A0082 diluted to 1/200) for identification of endothelial cells and blood vessel integrity. After incubation sections were washed 3×4 min in PBS, 0.05% Tween20, 0.1% BSA and incubated with appropriate secondary antibodies, goat anti-mouse alexa 488-conjugated antibody (A11017 diluted to 1/250, MoBiTec, Göttingen, Germany) or donkey anti-rabbit Cy3-conjugated antibody (711-067-103 diluted to 1/500, Dianova, Hamburg Germany).

Finally, sections were covered with Vectashield antifade Solution (Vector, Germany) containing 4′,6-diamidino-2-phenylindole (DAPI) as nuclear counterstain. Preparations were analyzed using a Zeiss Axioplan 2 fluorescence microscope equipped with the ISIS imaging system and appropriate fluorescent filter sets (MetaSystems, Altlussheim, Germany).

Regarding ASCs *in situ* localization, three days after each grafting specific biopsies centred on the injection plot of cells (10^6^ ASCs in each plot) were performed. In these cases ASCs were previously labelled by an overnight incubation with fluorescent quantum dots (Q-dots; Q-tracker 705, Invitrogen Life Technologies, Carlsbad, New-Mexico, US) to allow their delayed histological tracking.

### Statistical analysis

For the clinical study, comparisons in each group were performed using a Friedman RM Anova on ranks test or a Wilcoxon signed rank test for paired samples analysis (Statistica Software). The two animal groups were compared using a non parametric Mann-Whitney rank sum test. For the complimentary *in vitro* study we used a Wilcoxon matched pair test. A *p* value<0.05 was considered significant.

## Results

### Characterization of ASCs

**Figure 2:**
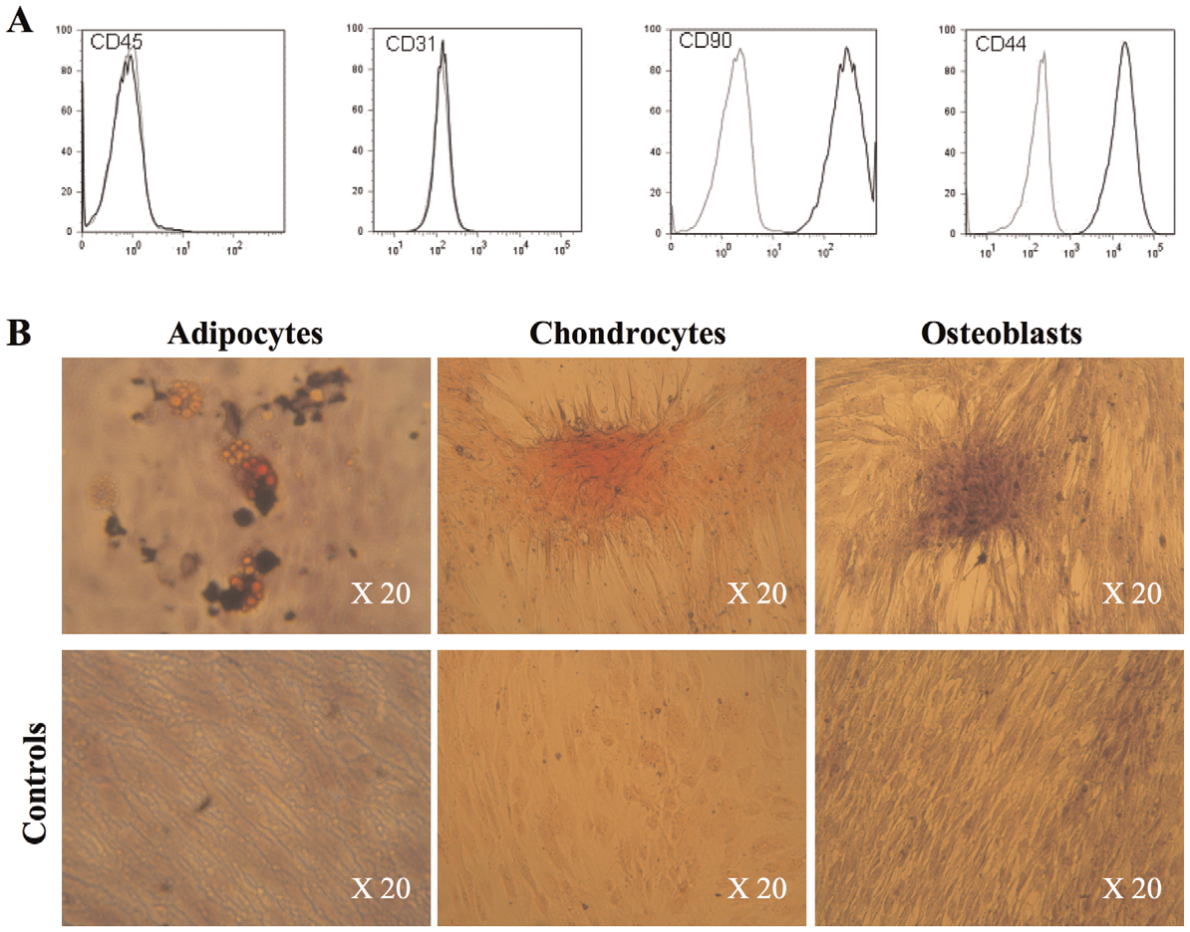
Characterization of ASCs. (**A**): Flow cytometric analysis after the 3^rd^ passage of porcine ASCs labelled with FITC- or PE-conjugated control isotype IgG (bold curves) or antibodies against indicated cell surface proteins (dark curves). (**B**): Differentiation of ASCs cultured in specific media.

Flow cytometry analysis of prepared ASCs showed that they expressed the typical surface antigens CD90 and CD44, but were negative for pan-hematopoietic CD45 and CD31 antigens (Fig. 2A). When cultured in adipogenic, chondrogenic or osteogenic medium, they differentiated into adipocytes (accumulation of lipid vesicles), chondrocytes (proteoglycans) and osteocytes (alkaline phosphatases) (Fig. 2B).

### Clinical evolution in controls

**Figure 3:**
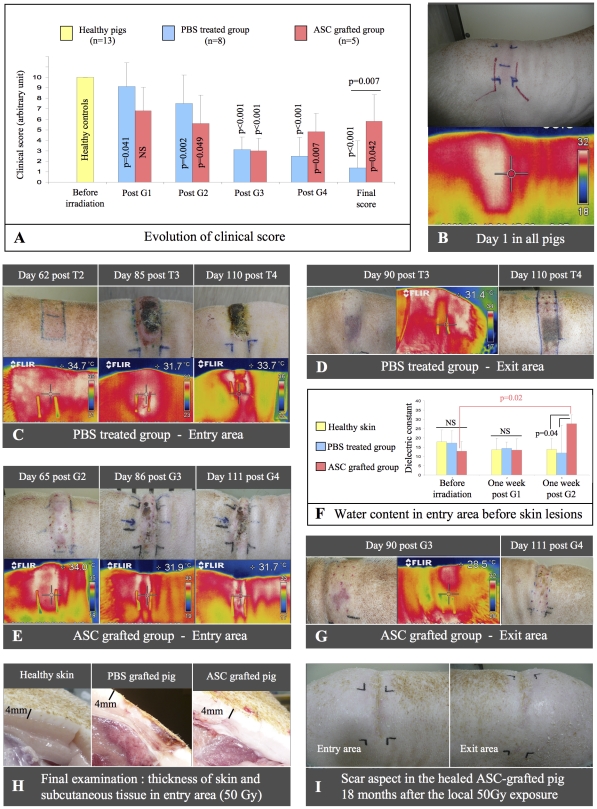
ASCs favoured wound healing after local irradiation. Evolution of clinical score (**A**). Clinic and thermal camera imaging from representative animals on day 1 (**B**), then in the entry/exit area of PBS-treated (**C/D**) or ASC-grafted minipigs (**E/G**). Water content in entry area skin (**F**). Final examination of skin thickness and subcutaneous tissue (**H**). Complete burn healing in one ASC-grafted minipig (**I**).

All controls exhibited a similar clinical evolution (Fig. 3A) in accordance with literature [2], [10], [11]. There was a transient reddening of the lumbar exposed area, which was confirmed by a superficial skin hyperthermia (Fig. 3B). A progressive hair loss was noted during 6 weeks post exposure, leading to a complete alopecia after two months. On about day 65, just before the third control PBS injection, the thermal camera imaging revealed a superficial skin hyperthermia in the entry area that was quickly followed by the swelling of the muscles and the distension of the cutaneous layer above the exposed tissues (Fig. 3C). This was followed by an extensive centrifugal moist skin desquamation, resulting in a thick scab over the entry area at day 76. At the same time, a cold purple spot due to vasculature failure developed in the 20–25 Gy exit area (Fig. 3D). Local intensive pain required morphine-based products after three months up to euthanasia of the animals. Finally, the skin wounds in both entry and exit areas progressively displayed extensive skin necrosis (from day 91) determining the end-point of the study on about day 119 after irradiation for 6 animals, and on days 124 and 140 for the two remainings. Necropsy confirmed that the lesions were limited to the irradiated tissues behind the transversal epiphysis of the lumbar vertebrae: skin necrosis, thinning of the hypodermis (Fig. 3H), and hemorrhagic lesions or hyaline degeneration in the muscles were locally observed.

### ASCs injection favours clinical wound healing after local irradiation

Each ASC injection in the damaged area induced a transient and painful localized inflammation for several days more severe than what we previously observed after bone marrow-mesenchymal stem cell (BM-MSC) injections [8]. The entry area displayed significant muscular swelling and thin scab lesions, which appeared significantly earlier than in controls (day 42±6 versus day 70±15, p = 0.003) (Fig. 3E) and before the second graft in two minipigs. Interestingly, the dielectric constant of the entire skin, which represents the water content of the whole dermis and subcutaneous fat, was significantly increased in ASC-treated minipigs between the second and the third grafts (p = 0.021) (Fig. 3F). In the exit area, a cold purple wound developed at the same time than in non-grafted controls (Fig. 3G). The irradiation-induced wounds necessitated an earlier analgesic treatment in grafted animals than in controls (day 81 versus day 89) and the treatment duration appeared longer (30 days versus 18 days) which may relate to the ASC-induced inflammation. Wound healing was observed in four out of five ASC-grafted animals at days 130±28 (range days 106 to 160). Local complete healing was observed in two grafted pigs, with disappearance of intercurrent inflammatory processes, pathognomonic for radiation burns, and total reepithelialisation (Fig. 3A) which persisted for more than 18 months post-exposure in one pig checked for long term follow up (Fig. 3I). The major analgesic treatment was not required in this animal in which pain was limited and could be dismissed after 6 weeks in the second one. Finally the reduced subcutaneous damage (Fig. 3H) and diminished delayed complications such as neurologic damage (confirmed after necropsy) accounted for a significantly extended survival time in the ASC-treated group in comparison with controls (day 139±16 and day 118±11 respectively, p = 0,016).

### ASC engraftment in the irradiated skin

**Figure 4:**
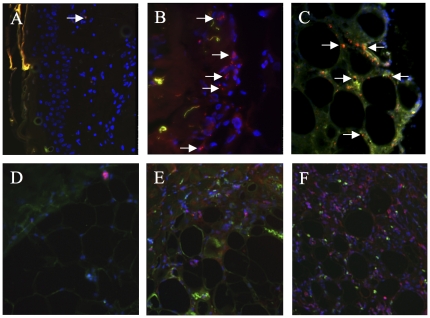
Engraftment of ASCs into the irradiated skin. Location of transplanted ASCs (in red) in the epidermis (A), in the dermis (B), and in the subcutis (C) of skin biopsies centred on injection plots, performed three days after the 3rd graft, on day 70. Brightly red fluorescing quantum dots marked ASCs (arrows) around blue nuclei (DAPI) near the cutis/subcutis border and among adipose tissue. The immunostaining of CD3 and lambda light chains (in pink) in the subcutis of healthy (D), PBS-treated (F), and ASC-grafted (F) skin underlined the infiltration by T and B lymphocytes three days after the ASC graft. Magnification: ×100.”

Three days after ASC grafting, Q-dot labelled ASCs were detected in the skin by epifluorescence microscopy. Grafted cells accumulated at the dermis/subcutis barrier (Fig. 4) where they had attracted numerous immune cells as reflected by an increased CD3 (T lymphocytes) and λ light chain (B lymphocytes) labelling relative to non-grafted control.

### ASCs enhanced reepithelialisation after irradiation

**Figure 5:**
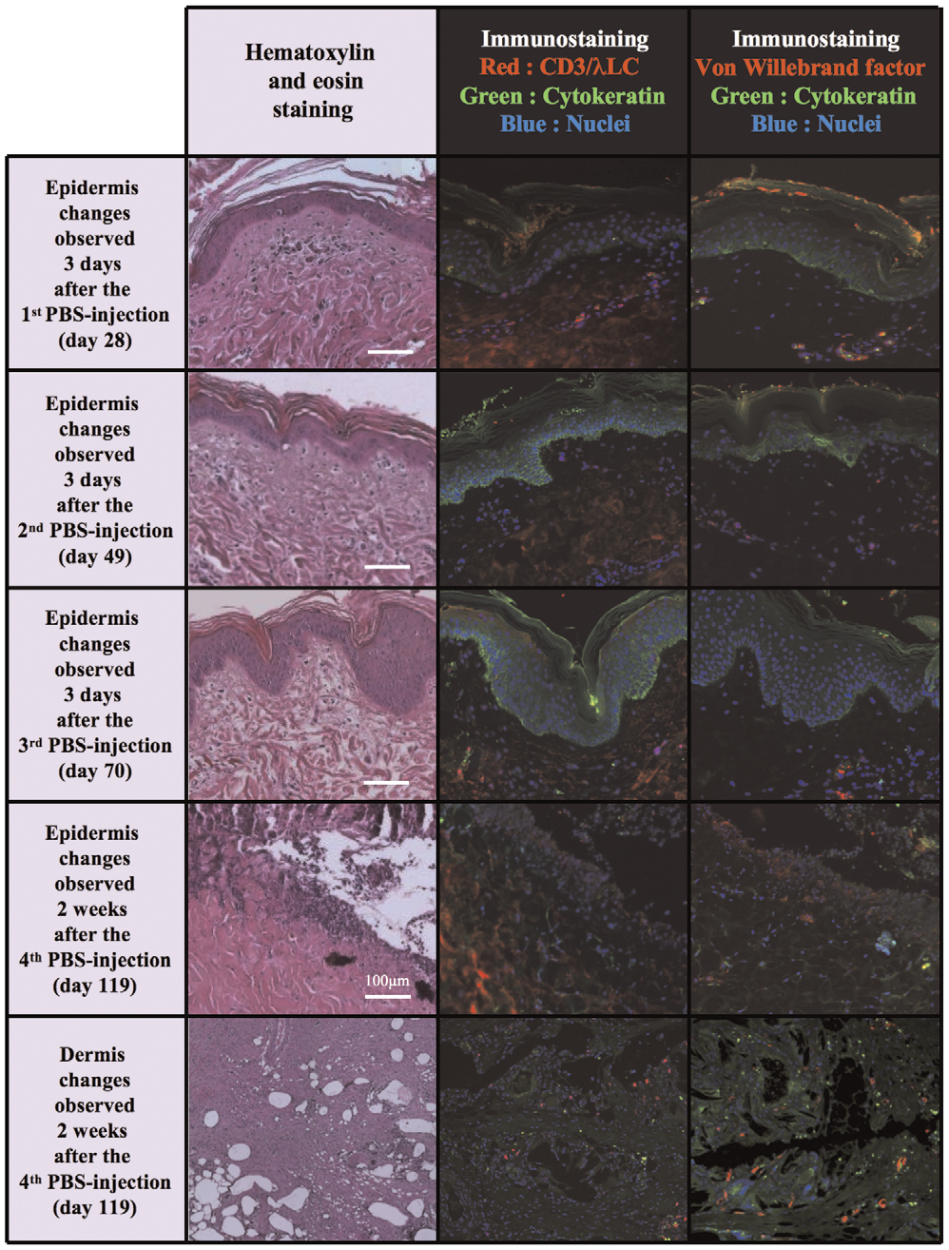
Epidermal and dermal radio-induced injuries in the skin of PBS-treated minipigs. Kinetic study from four iterative biopsies performed few days after each PBS-treatment in representative controls (magnification ×100). Progressive disorganisation of the epidermal layers, keratinocytes degeneration, vacuolation and barrier disruption. Numerous dermal cavities two weeks after the 4^th^ PBS-treatment.

**Figure 6:**
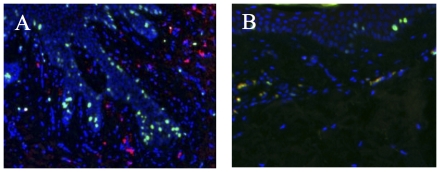
Proliferation of keratinocytes in grafted animals. After the third graft, the proliferation of keratinocytes in the epidermis-dermis junction is revealed by the Ki67 immunostaining (in green) in the irradiated skin of grafted animals (A) versus controls (B).

In PBS-treated control minipigs, the intense local irradiation exposure induced the formation of a scabby lesion of the skin three months post irradiation, that was progressively evolving into superficial necrosis (Fig. 5). The HE staining confirmed the clinical observations, with a progressive disorganisation of the epidermal layers, which was evident from the second month onwards after exposure. Pan-cytokeratin immunofluorescence revealed the degeneration of some keratinocytes as soon as day 49 after exposure, leading to vacuolation and the disruption of barrier function. At about day 70 (PBS-treatment 3) keratinocytes underwent proliferation as revealed by Ki67 staining (Fig. 6). Nevertheless, radio-induced lesions in control pigs developed into epidermal disorganization after four months, sometimes with complete destruction of dermo-epidermal junction and abrasion of epidermis.

**Figure 7:**
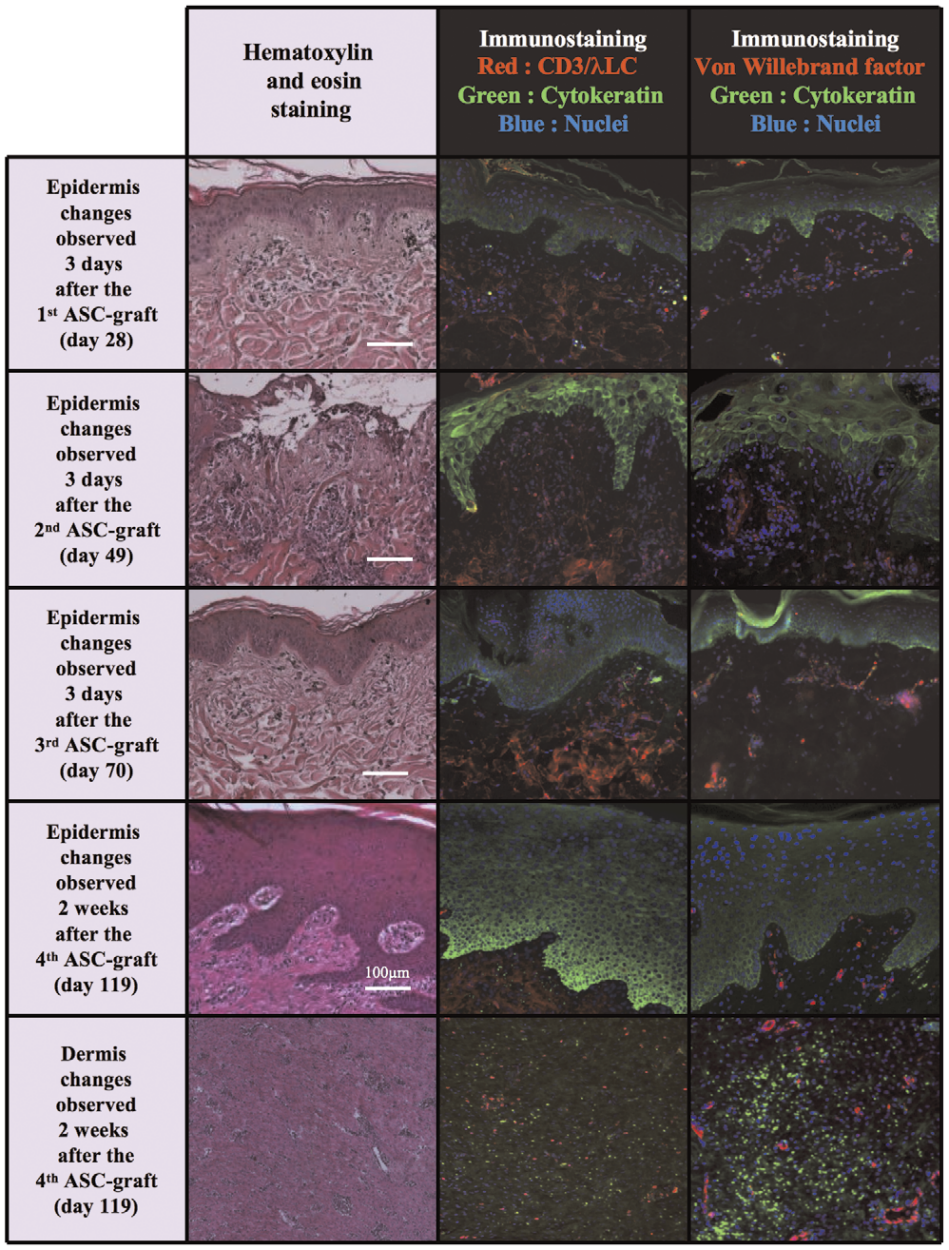
After local irradiation ASCs favoured reepithelialisation and enhanced lymphocyte infiltration and angiogenesis in dermis. Kinetic study from four iterative biopsies performed few days after each ASC-graft in representative animals (magnification in the original ×100). Early cleaning of damaged epidermis was followed by complete epidermis recovery/hyperproliferative keratinocyte activity. Two weeks after the 4^th^ ASC-graft, early T and B lymphocyte infiltration in dermis near the subcutis, with a strong increase in vascularisation.

In contrast, four out of five ASC-grafted animals exhibited a sustained epidermis recovery with a multilayered appearance similar to healthy skin. In the fifth minipig, the complete would healing could not be observed in the long term because its euthanasia was imposed for ethical reasons during the fifth month after exposure, that is before any complete skin evolution, because of a recurrent pain refractory to all analgesic treatment. In the four other ASC-grafted minipigs, cleansing of the damaged epidermis appeared earlier than in non-grafted controls and images of detersion were rapidly observed after the second graft (Fig. 7). The immunohistological analysis of cytokeratin expression showed a complete epidermis recovery, sometimes associated with a strong hyperproliferative keratinocyte activity leading to surnumerous cell layers in the entry area, revealed after DAPI staining of the cell nuclei, as shown in epidermis sections sampled after the fourth graft (Fig. 7).

### ASCs favoured lymphocyte infiltration in irradiated dermis

In controls, the dermis at the entry area level was still heavily damaged at the end of the study (>118±11 days) with the formation of numerous cavities and vessels of varying size which was associated with a progressive lymphocyte infiltration (Fig. 5). In ASC-grafted animals, the lymphocyte infiltration occurred earlier and was noticeable already after the second treatment (Fig. 7). In one of the grafted minipigs, three weeks after the last ASC injection (on day 119 i.e. after the 4^th^ graft), a strong specific CD3 and λ light chain immunohistological staining revealed a T and B lymphocyte infiltration especially near the subcutis (Fig. 7).

### ASC secretory activity stimulates *in vivo* angiogenesis

**Figure 8:**
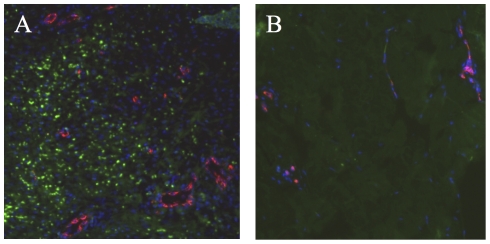
ASC grafting stimulated cutaneous vascularization. The red immunofluorescent staining of Von Willebrand factor revealed the increase of the vasculature in the dermis of the ASC-grafted minipig (A) in comparison with PBS-treated control (B), two weeks after the fourth treatment (day119). Magnification: ×100.

In parallel to lymphocyte infiltration, a strong increase in vascularization was observed by microscopy after the fourth graft in this ASC-grafted minipig (Fig. 8). When wound healing was achieved (day 119 to day 162 evaluations), vascular proliferation was absent in the other grafted animals.

## Discussion

Here we report the benefit of local autologous ASC grafting to prevent/cure CRS in a large animal model close to human. Wound healing was observed in 4 out of 5 treated animals whereas all PBS-injected controls exhibited a detrimental clinical evolution. We used a Göttingen minipig® CRS model which we previously showed to be relevant to the human accident situation of large area ionizing radiation exposure [8].

In this study we have tested an early graft schedule for ASCs which were injected in the irradiated area before the onset of local damages in controls. Clinical evolution was characterized by a transient local inflammation (clinic and dermis/subcutaneous fat tissue water content) not observed in our preliminary study with BM-MSCs [8] and in CRS patients given autologous BM-MSCs who exhibited a significant reduction of pain. This may represent a limit of the model or could be related to the specific graft cell content or to the earlier graft schedule than applied in published cases. Interestingly the subsequent muscular swelling and thin scab lesions that appeared earlier than in sham treated control pigs were followed by wound healing in four out of five ASC-grafted animals (complete in two of them). This contrasted with the irreversible evolution towards local necrosis in the controls. Histological analysis confirmed the clinic and highlighted a significant reepithelialisation as well as the stimulation of neo-angiogenesis in grafted animals. After autopsy, the thinning of dermis and subcutis appeared limited.

In our study, grafted animal exhibited a strong reepithelialisation. This is in accordance with Wu et al. who showed, using a mouse excisional wound splinting plus BM-MSC grafting model, that MSCs significantly accelerated wound healing in normal and diabetic mice and that BM-MSCs contributed to keratinocytes and dermal appendages [5]. Confocal microscopy showed cytokeratin-positive MSCs appearing in epidermis and dermis and forming nodules that resembled early swear or sebaceous gland. No cell fusion was observed. This was also reported by Ebrahimian et al. who used a biopunch plus local irradiation and ASC grafting model [12]. Here we were unable to localize ASCs near or inside the epidermis using the Q-dot technology. This could reflect a limit of this cell labelling strategy or alternatively be related to the difference in term of methods (no skin excision in our study), time of examination (3 days in place of 7 days post grafting) or animal model, or dilution of the cell label by multiple divisions. Thus our study did not support the transdifferentiation potential [12], [13], and reepithelialisation is likely to occur as a consequence of paracrine process which may involve angiopoietin-related growth factor release [14]. This wound healing process may be cell-specific as it was not observed following autologous BM-mononuclear cells injection which we checked in preliminary experiments. Regarding the postulated pro-angiogenic potential of ASCs, our results are in accordance with Ebrahimian et al. who reported in a mouse model of CRS a transient increase of angiogenesis following cell injection [12]. We clearly observed such a pro-angiogenic process in one of the ASC-grafted minipigs on day 119. Interestingly the Q-dot analysis clearly indicated that at this time ASCs were massively located at the deeper level of dermis, near the subcutis. Importantly a noticeable co-localization of immune cells in the neovascularized dermis was observed which suggests the importance of the recruitment and the activation of lymphoid cells and subsequent secretions in this context [15]. Thus further studies are required to deeper understand the CRS wound healing process but our study suggests that ASCs may have secreted pro-angiopoitic or other factors, while differentiation and/or integration, especially in vascular structures, likely played a limited if any role [16].

Regarding clinical use, the 18 months follow up of one minipig suggests the safety of ASC grafting [17]. The ASC dose schedule we used (1×10^6^ ASCs per cm^2^, four iterative grafting) was non-toxic and led to improved wound healing. However the feasibility of the early grafting strategy in humans will depend on the availability of large amounts of ASCs in the days following the hospitalization meaning allogeneic stem cells banking. Further studies are required to confirm the debated therapeutic potential of such grafts. This study also indicated that final morbidity highly depended of underlying tissue damages which required specific treatment. Another approach to severe CRS would consist in the application of artificial dermis substitutes colonized with multipotent/angiogenic stem cells when excision is required. Further studies will also explore the therapeutic potential of allogeneic and *ex-vivo* manipulated ASCs in this context [18].
